# Iron Deficiency without Anemia Decreases Physical Endurance and Mitochondrial Complex I Activity of Oxidative Skeletal Muscle in the Mouse

**DOI:** 10.3390/nu13041056

**Published:** 2021-03-24

**Authors:** Emmanuel Rineau, Naïg Gueguen, Vincent Procaccio, Franck Geneviève, Pascal Reynier, Daniel Henrion, Sigismond Lasocki

**Affiliations:** 1 MitoVasc Institut, UMR CNRS 6015—INSERM 1083, University of Angers, 49100 Angers, France; NaGueguen@chu-angers.fr (N.G.); vincent.procaccio@univ-angers.fr (V.P.); pascal.reynier@univ-angers.fr (P.R.); daniel.henrion@univ-angers.fr (D.H.); silasocki@chu-angers.fr (S.L.); 2 Department of Anesthesia and Critical Care, University Hospital of Angers, 49100 Angers, France; 3 Department of Biochemistry and Genetics, University Hospital of Angers, 49100 Angers, France; 4 Department of Hematology, University Hospital of Angers, 49100 Angers, France; frgenevieve@chu-angers.fr

**Keywords:** iron deficiency, striated skeletal muscle, physical capacity, fatigue, mitochondrial metabolism, complex I

## Abstract

Iron deficiency (ID), with or without anemia, is responsible for physical fatigue. This effect may be linked to an alteration of mitochondrial metabolism. Our aim was to assess the impact of ID on skeletal striated muscle mitochondrial metabolism. Iron-deficient non-anemic mice, obtained using a bloodletting followed by a low-iron diet for three weeks, were compared to control mice. Endurance was assessed using a one-hour submaximal exercise on a Rotarod device and activities of mitochondrial complexes I and IV were measured by spectrophotometry on two types of skeletal striated muscles, the soleus and the quadriceps. As expected, ID mice displayed hematologic markers of ID and reduced iron stores, although none of them were anemic. In ID mice, endurance was significantly reduced and activity of the respiratory chain complex I, normalized to citrate synthase activity, was significantly reduced in the soleus muscle but not in the quadriceps. Complex IV activities were not significantly different, neither in the soleus nor in the quadriceps. We conclude that ID without anemia is responsible for impaired mitochondrial complex I activity in skeletal muscles with predominant oxidative metabolism. These results bring pathophysiological support to explain the improved physical activity observed when correcting ID in human. Further studies are needed to explore the mechanisms underlying this decrease in complex I activity and to assess the role of iron therapy on muscle mitochondrial metabolism.

## 1. Introduction

Fatigue is one of the main symptoms of iron deficiency (ID), even in absence of anemia. Iron deficiency-related fatigue may present as a mental or as a physical fatigue, affecting mainly endurance, and correction of ID has been shown to improve both fatigue and physical activity [1,2,3,4,5,6,7,8]. In some populations such as heart failure patients, ID, likely through muscle fatigue, is also responsible for a deterioration of the quality of life, a worsening of dyspnea and a worsening of the prognosis of heart failure [9,10,11,12]. Interestingly, intravenous iron therapy has also been shown to improve these parameters in heart failure patients, even in the absence of anemia [13,14,15,16,17].

Mechanisms linking ID to muscle fatigue are still poorly understood. The main hypothesis is that ID is responsible for an alteration of mitochondrial muscle metabolism, iron being present in both iron-sulfur centers and in cytochromes of the mitochondrial respiratory chain. Animal studies performed many years ago reported various quantitative and functional anomalies in mitochondrial respiratory chain enzymatic complexes in both skeletal muscles and heart [18,19,20,21,22]. However, the proper role of iron deficiency was difficult to evaluate in these studies, because of a severe anemia systematically observed in their animal models.

We recently developed a mouse model of ID without anemia, in which we assessed the impact of iron deficiency on mitochondrial metabolism in the myocardium [23]. In this model, the mitochondrial respiratory chain complex I activity was decreased in cardiomyocytes, which might explain, at least in part, a significant reduction of left ventricular function and in physical capacity during endurance exercises. However, ID may also impact the mitochondrial metabolism of peripheral skeletal muscle, as recently reported [24,25]. The aim of this study was to assess the impact of ID on the respiratory chain complex I activity of two types of striated muscle fibers in a mouse model of ID without anemia.

## 2. Materials and Methods

### 2.1. Animals and Ethics

All experiments were performed in accordance with the guidelines from Directive 2010/63/EU of the European Parliament on the protection of animals used for scientific purposes (laboratory authorization of the laboratory #00577). The protocol was approved by the Ethics Committee in animal experimentation of Pays de la Loire and by the French Ministry of Higher Education and Research (APAFiS #6780).

Male C57BL/6 mice (Janvier, Le Genest St Isle, France) were used for all experiments and all of them were eight-week-old at the start of the experiments. Mice were four per cage, housed in a temperature-controlled room (21 °C) with a 12 h/12 h light-dark cycle. They were fed either with an iron-deficient diet or a normal diet depending on their group and had access to tap water *ad libitum*.

The mouse model of ID without anemia (ID group) was obtained as previously described: on day 1, mice had a 250–300 µL blood withdrawal performed using a retro-orbital collection with a calibrated heparinized capillary tube. Mice were then immediately fed with an iron-depleted diet (C1038 pellets containing 6 mg iron/kg, Genestil SA, Royaucourt, France) for three weeks [23]. As previously shown, mice were considered to have an ID without anemia on the last day of the third week (Day 21) [23]. Control group mice (C group) were fed with a normal diet (M25 pellets containing 150 mg iron/kg, Special Diets Services, France) during the whole study.

Blood withdrawal and sampling were performed under inhaled anesthesia with isoflurane 2% and all efforts were made to minimize suffering. Euthanasia of the animals was made by cervical dislocation, under inhaled anesthesia with isoflurane 2% too, in order to avoid the deleterious effects of CO_2_ on mitochondrial metabolism.

### 2.2. Experimental Design

Each group (ID and C) included eight mice. On day 0 and day 21, mice performed the physical exercises on a Rotarod device. The mice were euthanized on day 21 after the physical exercises and a blood sample, the liver, the spleen and muscle samples of quadriceps and soleus were taken. All muscles were immediately frozen in liquid nitrogen after sampling and stored at −80 °C until analyses.

### 2.3. Physical Tests

We used a Rotarod device to assess physical capacities of the animals, as already described [23,26]. The Rotarod is a device with a 3 cm diameter cylinder on which mice were individually placed. The cylinder rotated at a progressive acceleration speed followed by a stable speed until the fall of the mouse that made stop the cylinder. The time without falling and the falling speed were automatically recorded (HARotarod software version 1.40) and the calculation of the distance performed by the animals during the total exercise time was done.

The day before the test, the mice were trained to the device by performing an exercise with a constant acceleration speed of 10 to 20 rpm for 180 s followed by a constant speed of 20 rpm. Each time the mice fell, they were immediately put back on the cylinder which restarted its rotation with the same acceleration from 10 to 20 rpm. This training exercise was stopped after 15 min of running on the cylinder in total.

We assessed endurance of the animals over 1 h using two consecutive 30-min tests. On the day of the test (Day 0), the protocol consisted of a first test (“test 20”) at a constant acceleration speed of 10 to 20 rpm for 180 s followed by a constant speed of 20 rpm. The test was stopped after 30 min of exercise and was immediately followed by a second test at a constant acceleration speed of 10 to 30 rpm for 180 s followed by a constant speed of 30 rpm. This test was also stopped after 30 min of exercise.

### 2.4. Hematological Parameters

Hemoglobin concentration (Hb), hematocrit (Ht), mean corpuscular volume (MCV), mean corpuscular hemoglobin (MCH), mean corpuscular hemoglobin concentration (MCHC), reticulocyte count, reticulocyte hemoglobin content (RetHb) and percentage of hypochromic red blood cells (% Hypo RBC) were measured using a hematological automate Sysmex XE-5000 (Sysmex France, Villepinte, France) on the blood samples obtained on Day 21.

### 2.5. Tissue Iron Content

Splenic and liver iron contents were measured after tissue digestion by trichloroacetic acid, hydrochloric acid and thioglycolic acid, using the iron quantification by the Ferene method on a biochemical automate ARCHITECT c16000 (Abbott France, Rungis, France), as previously described [23,27].

### 2.6. Mitochondrial Enzymatic Activities

Enzymatic activities were measured in two types of skeletal striated muscle: the quadriceps muscle, which has both oxidative and glycolytic metabolisms, and the soleus, which is mainly oxidative.

Complex I, primarily affected in the heart in our previous study, and complex IV activities were measured at 37 °C with a UVmc2 spectrophotometer (SAFAS, Monaco), according to standard methods [28]. Results were normalized to the citrate synthase activity, an enzyme of the Krebs cycle reflecting the mitochondria content.

Post-nuclear muscle homogenates were prepared at 4°C. The muscle isolation buffer used was composed of 220 mM mannitol, 75 mM saccharose, 10 mM Tris, 1 mM EGTA adjusted to pH 7.2. The muscle sample was rinsed in the isolation buffer and transferred into a glass tube containing 10 times its weight of the same buffer or 20 times its weight for the soleus. The muscle was homogenized at 1000 rpm with a Potter-Elvehjem PTFE and was centrifuged at 650 g for 20 min. The supernatant was sampled, and the operation was repeated on the pellet. Both supernatants were combined to constitute the post-nuclear muscle homogenate, which was used immediately.

Briefly, NADH ubiquinone reductase (complex I) activity was assayed in KH_2_PO_4_ buffer (50 mM, pH 7.5), containing 3.75 mg/mL fatty acid-free BSA and 0.1 mM decylubiquinone. 10 mM NADH was added to initiate the reaction. Parallel measurements in presence of rotenone (2.5 μM) were used to determine the background rate. Cytochrome c oxidase (complex IV) activity was assayed in a 92–97% reduced cytochrome c solution. Citrate synthase (CS) activity was assayed in a 0.15 mM DTNB, 0.1% Triton, 0.5 mM oxaloacetic acid and 0.3 mM acetyl-CoA solution. Absorbance changes due to the respective substrate conversions were monitored at 340 nm for complex I, 550 nm for complex IV, and 412 nm for CS. Enzymes activities of complex I and IV, expressed as nmol substrate/min/mg of proteins using the Beer Lambert’s law, were then normalized to the CS activity. Reagents were purchased from Sigma-Aldrich (Lyon, France) and NADH from Roche Applied Sciences (Lyon, France).

### 2.7. Statistical Analysis

Data are reported as medians [interquartiles 25–75%] or numbers (percentages). Categorical and numerical data were compared using the Fisher’s exact test and the Mann–Whitney test respectively. All tests were two-tailed and a p-value less than 0.05 was considered significant. Analyses were performed the software JMP (SAS Institute, Inc., Cary, NC, USA).

## 3. Results

### 3.1. Description of the Mouse Model of ID

**Figure 1:**
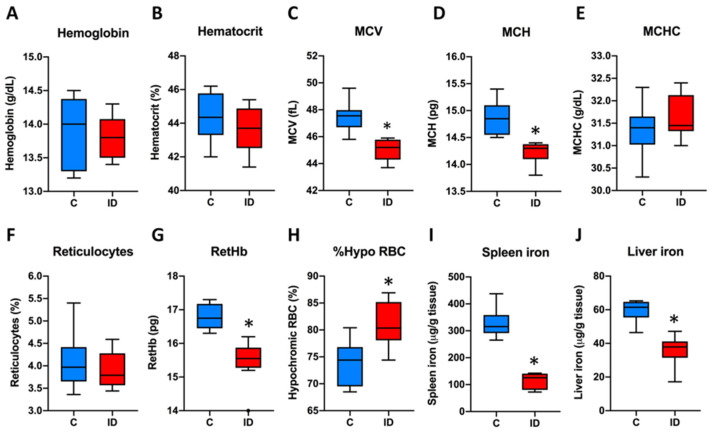
Hematological parameters and iron stores measured on Day 21. (**A**) Hemoglobin concentration; (**B**) hematocrit; (**C**) mean corpuscular volume; (**D**) mean corpuscular hemoglobin; (**E**) mean corpuscular hemoglobin concentration; (**F**) reticulocyte count; (**G**) reticulocyte hemoglobin content; (**H**) percentage of hypochromic red blood cells; (**I**) iron content in the spleen; (**J**) iron content in the liver. C (blue boxes), control group; ID (red boxes), ID group; N = 8 in each group. Box-plots represent medians, interquartile ranges and upper and lower values according to Tukey’s method. * *p* < 0.05, significantly different from control group.

As shown in Figure 1A,B, none of the mice were anemic, with hemoglobin and hematocrit levels greater than 13 g/dL and 40% respectively, without differences between ID and C mice groups. Conversely, the mice of the ID group had hematological signs of ID including a significant decrease in RetHb and a significant increase in % Hypo RBC (Figure 1C–H). Furthermore, tissue iron stores in the spleen and liver were significantly reduced (Figure 1I,J).

### 3.2. Animal Physical Endurance

**Figure 2:**
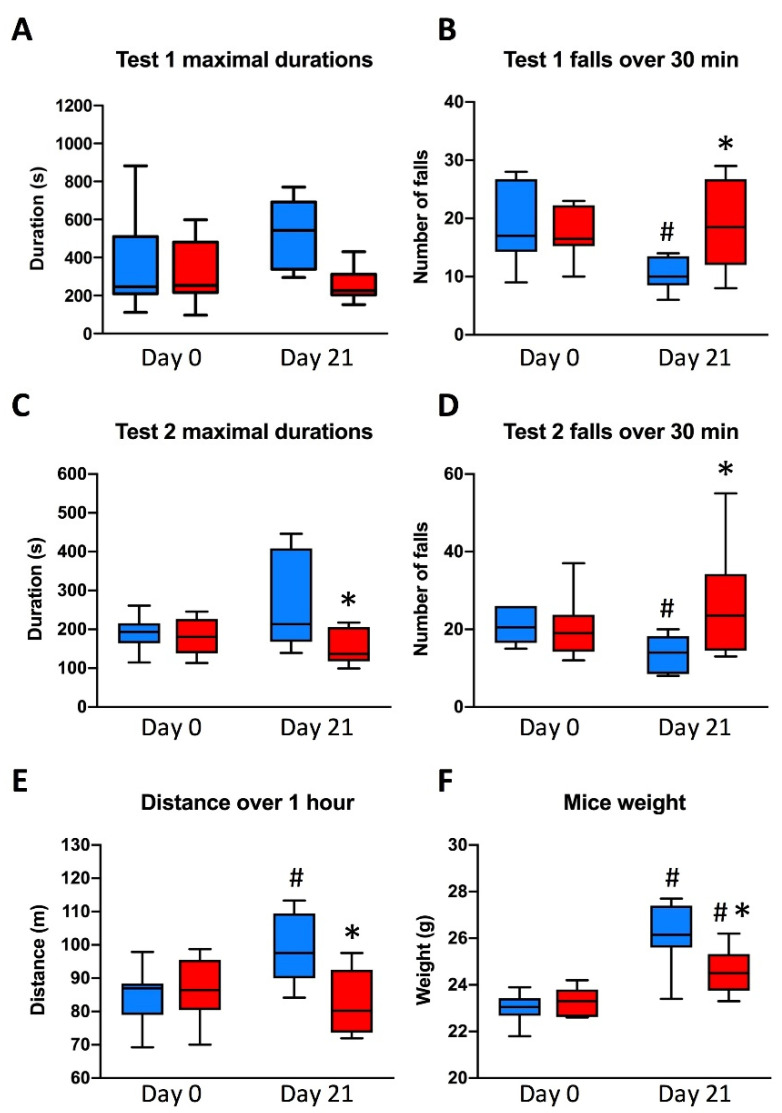
Endurance measured on Rotarod and mice body weights. (**A**) Maximal durations and (**B**) number of falls in test 1 (speed of 10 to 20 rpm for 180 s followed by a constant speed of 20 rpm), (**C**) maximal durations and (**D**) number of falls in test 2 (speed of 10 to 30 rpm for 180 s followed by a constant speed of 30 rpm), (**E**) distance over 1 h performed by mice, and (**F**) weight of mice. C (blue boxes), control group; ID (red boxes), ID group; N = 8 in each group. Box-plots represent medians, interquartile ranges and upper and lower values according to Tukey’s method. * *p* < 0.05 compared with control group on the same day; # *p* < 0.05 compared with the same group on Day 0.

The physical capacities of the animals were similar on Day 0 in both groups (Figure 2A–E). On Day 21, ID mice achieved significantly lower maximum times on Rotarod than ID mice on Day 0 and than control mice on Day 0 and Day 21 (Figure 2A–C). The number of falls per test was significantly higher in ID mice on Day 21 than in control mice (Figure 2B–D). Consequently, the distance performed over one hour was significantly reduced in ID than in Control mice (Figure 2E). Mice of the group C had improved their total distance compared to Day 0, although the mice of the group ID had not. We also observed that mice in the ID group had a significantly lower weight at week 3 than Control mice (Figure 2F).

### 3.3. Mitochondrial Enzymatic Activities

**Figure 3:**
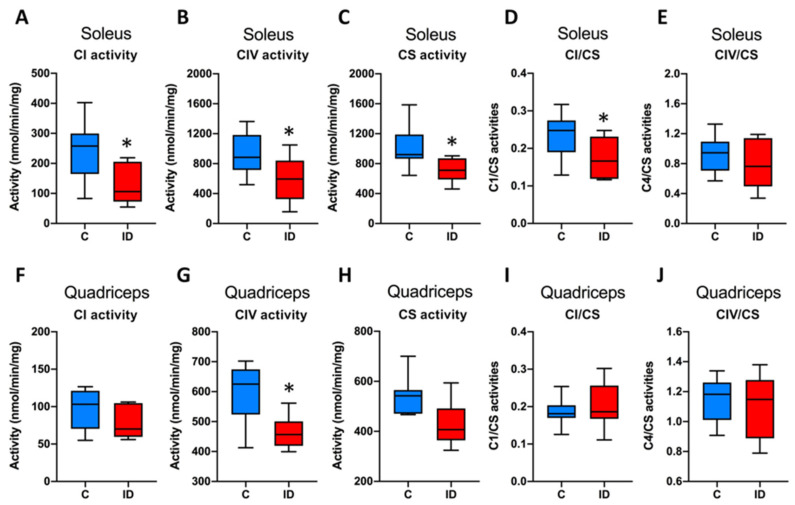
Mitochondrial enzymatic activities. Maximal enzymatic activities of complex I, complex IV, citrate synthase, were measured by spectrophotometry on muscle homogenates of soleus (**A**–**C**, respectively) and quadriceps (**F**–**H**, respectively). CI/CS and CIV/CS: specific activities of complexes I and IV normalized to the citrate synthase one (**D**,**E**: soleus muscle; **I**,**J**: quadriceps). C (blue boxes), control group; ID (red boxes), ID group; N = 8 in each group. Box-plots represent medians, interquartile ranges and upper and lower values according to Tukey’s method. * *p* < 0.05 compared with control group.

The maximal activities of complex I, complex IV and citrate synthase are shown in Figure 3. The activities of complexes I and IV, together with citrate synthase, were all significantly reduced in the soleus muscle of ID mice, suggesting a decrease in mitochondrial content. When the activities of complexes I and IV were normalized to those of citrate synthase, we observed a significant decrease in the activity of complex I in the soleus, without significant differences in activity for complex IV in the soleus and for complexes I and IV in the quadriceps.

## 4. Discussion

In this study, confirming that ID without anemia is associated with a decrease in endurance capacity, we showed a predominant impact of ID on complex I activity in oxidative skeletal muscle.

The impact of ID, independently of anemia, on physical capacities is difficult to measure. In our mouse model, we used long and submaximal exercises on a Rotarod device to assess endurance. Using this test, we observed a reduction in the distance achieved in one hour of about 15% in ID mice. Overall, previous animal studies found a decrease in both maximum oxygen consumption and endurance capacity [18,19,20,21,29]. However, in these studies evaluating physical capacities in ID animals, ID was accompanied by severe anemia, at least at some point of their experiments. Davies et al. showed in a mouse model of ID anemia that anemia mainly alters maximum oxygen consumption [19]. The increase in hemoglobin levels was indeed associated with an early improvement of maximum oxygen consumption after dietary iron repletion, while physical endurance was improved several days later. These results led the authors to suggest that the maximum oxygen consumption, and therefore the capacity to exercise with maximum effort, is mainly due to a reduction of hemoglobin level, whereas endurance seems rather dependent on the oxidative capacities of the muscle, i.e., on mitochondrial metabolism. Conversely, Willis et al. observed in rats fed with an iron-depleted diet a significant decrease in endurance several days before the fall in hemoglobin level and, after an iron dextran injection once the animals were anemic, a very fast improvement (beginning at the 15th hour) of the running time on a treadmill, i.e., before the increase in hemoglobin level [21]. In the model used in the present study, we had previously observed that ID without anemia affected endurance rather than short intense training exercises (e.g., forced swimming exercise) [23]. We confirm here the impact on endurance using a longer test (one hour in total).

In humans, psychic fatigue is a frequent symptom of ID without anemia, which is effectively corrected by iron therapy [1,2]. However, it has also been shown that physical capacities may be improved by iron repletion [30]. The benefit of ID correction, using intravenous iron, has also been demonstrated in heart failure patients [13,14,15,16,17,31]. Interestingly, recent data show that heart failure patients with ID have a greater depletion of phosphocreatine in the gastrocnemius muscle (measured by ^31^P magnetic resonance spectroscopy), which could partly explain muscle fatigue [24]. Likewise, Charles-Edwards et al. recently observed that iron therapy allows iron-deficient heart failure patients to decrease the regeneration time of skeletal muscle phosphocreatine [25]. Phosphocreatine is necessary during muscle contraction since it makes it possible to give a phosphate to ADP in order to regenerate ATP, and is itself regenerated at rest from ATP supplied by the respiratory chain and glycolysis. Thus, the impairment of physical capacities of these patients could be linked, at least in part, to the impairment of skeletal muscle function due to mitochondrial metabolism dysfunction.

In our mouse model of ID without anemia, we confirmed this impairment of skeletal muscle mitochondrial function in the soleus, but not in the quadriceps. This difference is probably explained by the fact that the soleus muscle is a muscle encompasses a large majority of oxidative type I and type IIA fibers (“red muscle”), while the quadriceps is a mixed muscle containing essentially types II, fast-twitch glycolytic, fibers but a reduced content in type IIA and type I, oxidative, fibers [32]. Type I and IIA fibers, rich in mitochondria, mainly use oxidative capacities of the cell, promoting a higher resistance to fatigue. Conversely, other type II fibers have a lower mitochondria density and have greater glycolytic than oxidative capacities, allowing a significant muscle strength but not a high resistance to fatigue. These particularities probably explain why endurance, which involves fibers with higher oxidative capacities, are mainly affected, in our model and in humans.

We observed in our model that mitochondrial complex I activity was reduced in skeletal muscle in presence of ID, as we had previously observed in the myocardium [23]. This reduction of activity of about 30% seems to be less important than in anemic animals [19], and, while previous studies using anemic animals showed decreased activities of the 4 complexes of the respiratory chain, it seems here to primarily affect the complex I. This result may be explained by the larger number of iron-sulfur centers located in complex I, in comparison with the three other complexes [33]. Iron atoms of iron-sulfur centers, present in complexes I (8–9 iron-sulfur centers), II (3 iron-sulfur centers) and III (1 iron-sulfur center), and iron atoms of heme cytochromes, present in complexes II (1 cytochrome), III (2 cytochromes) and IV (2 cytochromes), have an essential role for the function of the respiratory chain. Indeed, their capacity to change very easily from a ferrous (Fe^2+^) to a ferric (Fe^3+^) state facilitates the transfer of electrons required to induce the proton gradient across the inner mitochondrial membrane. Future studies should try to explain the mechanisms leading to the reduction of complex I activity. In our mouse model, we previously observed that this decrease seemed to be linked to a decrease in the overall amount of this complex in the myocardium [23]. The decrease in production of iron-sulfur centers could thus be responsible for a reduction in iron-sulfur centers, leading to a decrease in complex I assembly. However, other mechanisms have been suggested to explain the impact of ID on muscle mitochondrial metabolism, such as an early transition to anaerobic metabolism [34,35], the decrease in the transcription of genes coding for mitochondrial proteins mediated by the IRP/IRE intracellular iron homeostasis system [36], or even mitochondrial morphological alterations [37,38]. Interestingly, we also observed in our study a significant decrease in the activity of citrate synthase in the soleus muscle. The activity of this enzyme, present in the mitochondrial matrix, is commonly used as a reflect of mitochondrial mass [39]. The citrate synthase reduction therefore strongly suggests a decrease in mitochondrial content in soleus muscle, which could be linked to impaired mitochondrial biogenesis or turnover in response to iron ID.

Although these results need to be verified in humans, they open an important avenue for further studies aiming to explore the role of iron in muscle fatigue. A study is underway to evaluate mitochondrial metabolism of cardiomyocytes in cardiac surgery patients, according to their iron status (NCT03541213). Future studies could also focus on skeletal muscle function in patients with or without heart failure. They could allow the widening of iron treatment indications in non-anemic ID patients, as already recommended for heart failure patients [40], especially in situations where recovery of muscle function is required, as in the postoperative period.

This study has some limitations, related in part to the use of an animal model and a limited number of animals. Moreover, we made the choice to use only male mice to create the mouse model of ID without anemia, in order to avoid blood loss linked to the menstrual cycle, that could have been responsible for unwanted anemia. However, both male and female mice will have to be used in future research using this model of ID without anemia, in order to verify whether the impact of iron deficiency is different in female mice. In addition, while the use of a Rotarod device allowed us to evaluate endurance function at a submaximal effort, other functions, such as motivation, coordination or even animal learning are probably involved with the use of this device [41] and may be altered by ID [42,43]. Future human studies will have to use validated tests such as cycloergometer tests, the 6-min walk test, or fatigue and quality of life questionnaires. Finally, due to the small size of soleus muscles, we chose to measure maximal activities of the respiratory chain complexes by spectrophotometry only. Indeed, this method can detect a moderate decrease in an enzymatic complex activity, while oxygraphy only detects this decrease from a higher threshold of inhibition of the complex. However, oxygraphy analyzes will be necessary subsequently to assess the functionality of the whole respiratory chain, in our mouse model and in humans, to further explore the impact of the metabolic changes observed here.

## 5. Conclusions

In our murine model of ID without anemia, we confirmed that ID negatively affects endurance and is responsible for a decrease in complex I activity in the skeletal striated muscle with a predominant oxidative activity. These results bring new evidence for the rationale of physical fatigue associated to iron deficiency and the potential usefulness of iron therapy to prevent these symptoms.

## Data Availability

Data presented in this study are available in Raw data S1.

## Supplementary Materials

The following are available online at https://www.mdpi.com/2072-6643/13/4/1056/s1, Raw data S1.
